# Socio-ecological perspective of older age life expectancy: income, gender inequality, and financial crisis in Europe

**DOI:** 10.1186/s12992-017-0279-8

**Published:** 2017-08-18

**Authors:** Jong In Kim, Gukbin Kim

**Affiliations:** 1 Division of Social Welfare and Health Administration, Wonkwang University, Iksan, Republic of Korea; 2 Institute for Longevity Sciences, Wonkwang University, Iksan, Republic of Korea; 3 Global Management of Natural Resources, University College London (UCL), London, UK

**Keywords:** Gender inequality, Credit information, National income, Older age life expectancy

## Abstract

**Background:**

Population is aging rapidly in Europe. Older age life expectancy (OLE) can be influenced by country-level depth of credit information (DCI) as an indicator of financial crisis, gross national income (GNI) per capita, and gender inequality index (GII). These factors are key indicators of socio-ecological inequality. They can be used to develop strategies to reduce country-level health disparity. The objective of this study was to confirm the relationship between socio-ecological factors and OLE in Europe.

**Methods:**

Data were obtained from World Bank, WHO, and UN database for 34 Europe countries. Associations between socio-ecological factors and OLE were assessed with Pearson correlation coefficients and three regression models. These models assumed that appropriate changes in country-level strategies of healthy aging would produce changes in GNI per capital as personal perspective, GII in social environment perspective, and DCI in public policy perspective to implement socio-ecological changes. Hierarchal linear regression was used for final analysis.

**Results:**

Although OLE (women and men) had significant negative correlation with GII (gender inequality index, *r* = − 0.798, *p* = 0.001), it had positive correlations with GNI (gross national income per capita, *r* = 0.834, *p* = 0.001) and DCI (depth of credit information index, *r* = 0.704, *p* = 0.001) levels caused by financial crisis. Higher levels GNI and DCI but lower GII were found to be predictors of OLE (women and men) (R^2^ = 0.804, *p* < 0.001).

**Conclusions:**

Factors affecting older age life expectancy in Europe were identified from socio-ecological perspective. Socio-ecological indicators (GII, GNI, and DCI) in Europe appear to have a latent effect on OLE levels. Thus, country-level strategies of successful aging in Europe should target socio-ecological factors such as GII, GNI, and DCI value.

**Electronic supplementary material:**

The online version of this article (doi:10.1186/s12992-017-0279-8) contains supplementary material, which is available to authorized users.

## Background

The population is aging rapidly in Europe. The proportion of people aged 65 years and older is forecasted to increase from 14% in 2010 to 25% in 2050 [1]. People in nearly every part of Europe are living longer. However, their chance of spending these later years in good health and well-being varies within and between countries [1]. Although previous studies have determined country-level healthy life expectancy, health expenditure, and income [2, 3], country-level older age life expectancy associated with national financial crisis and gender inequality have been overlooked [3−6]. Thus, socio-ecological perspective of older age life expectancy needs to be explored to contribute to research and public policies for the elderly. No studies have reported older age life expectancy (OLE) by gross national income (GNI) per capita, gender inequality index (GII), and depth of credit information (DCI) caused by financial crisis in Europe countries from socio-ecological perspective.

The financial crisis in Europe has posed major threats to health and related systems [5]. Even though Greece, Spain, and Portugal have adopted strict fiscal austerity, their economies have continued to recede, placing considerable strain on their healthcare systems. Outbreaks of infectious diseases are increasingly common in these countries. Budget cuts have restricted access of people to healthcare [5]. Before accepting large cuts in public spending as a measure, it is important to compare the lack of evidence for such short-term fixes that have potentially dire repercussions on population health and welfare [7].

DCI index is an indicator of a financial crisis [8]. GNI per capita, GII, and DCI are key indicators of socio-ecological inequality. Older age life expectancy (OLE) can be influenced by country-level CNI, GII, and DCI. Indeed, DCI (sovereign credit rating indicator) GNI, and GII (indicators for standard of living) might be useful for developing ways to reduce health disparity.

Several studies have studied health inequalities and economic crises across countries [5, 7, 9–17] or national income and gender inequality in health inequalities [3, 18–21]. However, few studies have addressed the relationship between socio-ecological inequality indicators including GNI, GII, and DCI caused by financial crisis and OLE [5, 12, 13, 15]. A retrospective analysis of health level factors contributing to GNI, GII, and DCI may help identify key determinants of OLE in a country. The impact of OLE on national income, gender inequality, and financial crisis compared between countries can inform policy decisions regarding OLE leading to socio-ecological inequalities. Thus, the objective of this study was to determine correlations of OLE with GNI, GII, and DCI caused by financial crisis and compare them between countries from socio-ecological perspective.

In this study, three socio-ecological factors (GNI, GII, and DCI reflecting national income, gender inequality, and financial crisis, respectively) were assumed to be associated with older age life expectancy (OLE). The current study aimed to better understand the influence of OLE as an indicator of older age health disparity in 34 European countries by examining its contribution to national income, gender inequality, and financial crises. Socio-ecological inequality in countries with high value of OLE is expected to be smaller because of their higher GNI and DCI advantages but lower GII disadvantages.

Healthy aging is multifactorial and quantitative. It can be influenced by biological, psychosocial, and environmental factors [3, 18–26]. However, sociocultural components of a country-level OLE mentioned above have not been studied in relation to socio-ecological inequality indicators. Although studies have indicated that social and health level factors [3, 27–30] such as GNI, GII, and OLE [3, 18–21] can predict socio-ecological inequality, this study aims to confirm if these factors are affected by a country’s DCI. Furthermore, although studies have investigated the effect of health and economic crisis [5, 12, 15, 31, 32], the association between country-level OLE and GNI, GII, or DCI has not been reported. Finally, the claim that OLE might be influenced by country-level socio-ecological inequality factors has not been tested using indicators at national level across Europe. Thus, associations between country-level OLE and socio-ecological inequalities such as national income, gender inequality, and financial crisis were confirmed in this study.

## Methods

### Conceptual framework for OLE and socio-ecological perspective factors

The proposed conceptual framework depicting the effect of the socio-ecological indicators on OLE is shown in Additional file 1. OLE can be affected by socio-ecological indicators. The term ‘OLE’ refers to being physically active with preservation of functional capacity and socioeconomic wellbeing as exemplified through an active life in society without diseases [3, 19, 21, 22, 26]. Consequently, OLE inequality may be affected or controlled by socio-ecological environment and hereditary factors, although the latter was excluded from this study. Thus, macro-level socio-ecological inequality factors were focused on in this study [3, 19–21, 33].

Health promotion is determined by influences at multiple levels, including personal, community, and public policy factors from socio-ecological perspective [34]. Socio-ecological perspective in OLE may identify influences of personal, social environmental factors, and public policy on older adult’s health and individual behaviour. These factors might be human aggregate or characteristics of people and/or characteristics of the surrounding community and country [35]. Thus, this article proposed a socio-ecological inequality model for OLE focusing on the following: (1) personal perspective, (2) social environment such as human relations, and (3) public policy perspective as targets for health promotion [34, 36].

To implement socio-ecological changes, the model assumed that changes in national income per capita from personal perspective, changes in gender inequality of human relations from social environment perspective, and changes in the depth of credit information caused by financial crisis from public policy perspective would produce appropriate changes in country-level strategies of successful aging (Additional file 1). Thus, this study determined the association of elderly life expectancy (OLE) with gross national level income per capita (GNI) at personal level, gender inequality (GII) at socio-cultural level, and depth of credit information (DCI) on national finance in public policy at socio-ecological level to identify successful strategy for an aging society.

This study was a socio-ecological research that designed model affecting OLE according to personal level, social environment, and public policy. In other words, personal level reflects economic condition while social environment and public policy reflect gender discrimination level and national financial status, respectively. Personal level was set as gross national income per capita (GNI) because it had the most significant impact on medical expenditure of the elderly. Social environment was set as gender inequality (GII) considering that inequality between men and women could be an important stress in elderly of a modern society at socio-cultural level. Public policy was set as depth of credit information (DCI) in national finance condition because it might affect the health and welfare of the elderly. These selected indicators could be used in a model to predict factors that affect life expectancy of the elderly in each country in the long term. In other words, with a national socio-ecological analysis model, causal association and influences of factors on OLE could be predicted. Based on these results, strategies could be suggested to promote health policies and improve life expectancy of the elderly in an aging society (Additional file 1).

### Hypothesis and model setting

To examine associations between OLE levels and socio-ecological perspective indicators, models were developed to estimate income, gender, and financial crisis disparities in relation to each variable. Three socio-ecological models were developed considering the following: (1) GNI per capital from personal perspective, (2) GII from social environment perspective, and (3) DCI as financial crisis from public policy perspective. These models were used to depict the proposed framework for socio-ecological indicators on the basis of selected variables. Specifically, predictors of national income, gender inequality, and financial crisis disparities (i.e., GNI, GII, and DCI respectively) were used to create a combination model or a model comprising all three models. These variables were reflective of all selected socio-ecological inequality indicators. Thus, the relationship between OLE may differ according to national income, gender inequality, and financial crisis. From this model, it was hypothesized that increases in GII would result in corresponding decreases or increases in OLE. However, decreases in GNI and DCI would result in corresponding decreases or increase in OLE. Associations between these factors and OLE in three models were assessed using Pearson correlation coefficients and regression models [19, 21]. A regression analysis was run to determine whether each socio-ecological indicator was independently and significantly correlated with OLE. The following multivariate regression models were used to analyze there indicators: Model (1) for OLE (women & men), Y_1_ = a + bx_1_ + bx_2_ + bx_3_···· +ε, Model (2) for OLE (men), Y_2_ = a + bx_1_ + bx_2_ + bx_3_···· +ε, Model (3) for OLE (women): Y_3_ = a + bx_1_ + bx_2_ + bx_3_···· +ε, Where a was intercept term or constant, b was unknown parameter, ε was a random error term, x_1_ was GNI, x_2_ was GII, and x_3_ was DCI. Hierarchal linear regression was used for final analysis.

### Data collection and terminology

Data for OLE analysis were obtained from life expectancy survey conducted by the World Health Organization [37]. DCI and GNI data used for this study were adopted from World Development Indicators of the World Bank [8, 38]. Data for GII indicators were obtained from United Nations [39] datasets. Necessary permissions were obtained to publish these data since no personal data were presented.

The following factors were used from socio-ecological perspective: (1) OLE (Older age life expectancy, years) for 2000 ~ 2012. It was defined as the average number of years a person expected to live in ‘full health’, including years lived with less than full health owing to diseases and/or injuries [37]; (2) GNI (Gross national income per capita) for 2005 ~ 2015. It was defined as PPP (current international $) for 2005 ~ 2012 converted to international dollars using purchasing power parity rates [40]; (3) GII (gender inequality index, value: 0 = women and men equally, to 1 = women poorly) for 2000–2010. It was a composite measure reflecting inequality in achievements between women and men in three dimensions: reproductive health, empowerment, and labour market [39]; and (4) DCI (0 = low, to 6 = high) for 2004–2012. A score of 1 was assigned for each of eight features of credit bureau or credit registry or both [5, 38].

OLE at age of 60 years is derived from life tables. It is based on sex and age-specific death rates [37]. The estimated number of deaths in a life table and population by age group are aggregated in a given region in order to compute regional life tables [37]. The WHO uses a standard method as described above to estimate and project life tables for all member states using comparable data. This may lead to minor differences compared to official life tables prepared by member states themselves [19, 21]. With regard to year, OLE in this retrospective study reflected total women and men. OLE of women or men from 2000 to 2012 [OLE = (OLE in 2000 + OLE in 2012) / 2] [19, 21] was also analyzed.

DCI measures rules that might affect the scope, accessibility, and quality of credit information available through public and private credit registries. DCI is an index that ranges from 0 to 8, with higher values indicating availability of more credit information [8]. The index shows how DCI (0 = low to 6 = high) varies by country. In 2012, countries with the highest DCI value in Europe were the United Kingdom, Germany, and Lithuania with an average value of 6.00. However, Malta reported the lowest value of 0.00 [8].

GII measures gender inequalities in three important aspects of human development [41]. In other words, GII reflects inequality in achievements between women and men in three dimensions: reproductive health, empowerment, and labor market [18, 41, 42]. Thus, GII reflects women’s disadvantage in three dimensions for as many countries as possible with data of reasonable quality. It ranges from 0 (women and men fare equally) to 1 (women fare as poorly as possible in all measured dimensions) [18, 41, 42].

GNI per capita is based on purchasing power parity (PPP). PPP GNI is a gross national income (GNI) converted to international dollars using PPP rates [40]. Data are in current international dollars based on 2011 ICP round [40]. Thus, GNI in this retrospective study reflected the GNI from 2005 to 2012.

### Mean rate for time series data of OLE, GNI, GII, and DCI

To analyse socio-ecological inequalities, this study excluded countries with insufficient gender inequality or financial crisis information. Because OLE inequalities are based on cohorts with healthy lives, national income, gender inequality, and financial crisis indicators might have changed during the study period. Thus, examining the association between OLE and socio-ecological indicators would be appropriate. Time series data are available for GNI, GII, DCI, and OLE, allowing for more robust results. However, the present study has two comparability issues [3]: comparability between countries and comparability across time periods. These are accounted for in socio-ecological indicators. Because OLE level is a type of dynamic output indicator that represents morbidity improvement. It is closely related to country-level socio-ecological inequalities. Therefore, an equivalence indicator must be employed [3]. Thus, national income, gender inequality, and financial crisis levels were used as “mean rate of change index (MC)”. Because some countries experienced greater progress than others between 2000 and 2012, MC was used to supplement faults in such information. Specifically, MC considered both the present and changed value of indicators from 2000 to 2012. MC is the change in value of an indicator divided by time elapsed [3, 19, 43, 44]. Specifically, MC is the sum of the current value of socio-ecological indicator and its changed values from 2000 to 2012 divided by the number (N) of elapsed years: MC of national income indicators = [the value of indicators in 2005 + the value of indicators in 2012) / N]), MC of financial crisis indicators = [the value of indicators in 2004 + the value of indicators in 2012) / N], MC of gender inequality indicators = [the value of indicators in 2000 + the value indicators in 2010) / N] [3, 19, 21]. It was used as an estimate of time series data of socio-ecological inequality indicators. Indicators without sufficient time series data were excluded from this study.

## Results

### Disparity in OLE and socio-ecological inequality indicators

Results of descriptive statistics for OLE ranges across countries and socio-ecological inequality indicators values are summarized in Table 1. The mean GNI was $26,938 with a between-country disparity of $50,060. Mean DCI generally ranged from 1.51 in Slovenia to 6 in the United Kingdom. Mean GII was 0.186 with a between-country disparity of 0.38. OLE (Women and men) ranged from 17 years in the Russian Federation to 24 years in Europe (Iceland, Switzerland, France, Italy, and Spain), with a mean of 21 and 7 years in disparity between countries, respectively.

**Table 1:** Descriptive statistics of variable

Variable	N	Mean	SD^a^	Minimum	Maximum
OLE (women and men)	34	21.21	2.182	17	24
OLE (men)	34	18.92	2.591	13.5	23
OLE (women)	34	23	2.117	19	26.5
GNI	34	26,938	12,806	7355	57,415
GII	34	0.186	0.094	0.06	0.44
DCI	34	3.794	1.321	1.51	6

^a^Standard deviation

*GNI* Gross national income per capita, PPP (current international $), 2005–2012, *GII* Gender inequality index (value: 0 = women and men equally, to 1 = women poorly), 2000–2010, *DCI* Depth of credit information index caused by financial crisis, (0 = low, to 6 = high), 2004–2012, *OLE* older age life expectancy, at age 60 years, 2000–2012

### OLE prediction variables

Results of OLE, GII, and DCI indicators of socio-ecological in 34 European countries are shown in Table 2. Country-level OLE was correlated with GNI, GII, and DCI caused by financial crisis. Although OLE had positive correlations with GNI (*r* = 0.834, *p* = 0.001) and DCI (*r* = 0.704, *p* = 0.001), it showed significantly negative correlation with GII (*r* = −0.798, *p* = 0.001).

**Table 2:** Univariate variables for the OLE

Variable		Correlations Coefficient	T-Value	*P*-Value	R^2^
OLE (women and men)	GNI	0.834	8.52	0.001	0.69
	GII	−0.798	−7.52	0.001	0.64
	DCI	0.704	5.608	0.001	0.5
OLE (men)	GNI	0.798	7.481	0.001	0.64
	GII	−0.75	−6.47	0.001	0.57
	DCI	0.728	6.011	0.001	0.53
OLE (women)	GNI	0.803	7.613	0.001	0.64
	GII	−0.8	−7.6	0.001	0.64
	DCI	0.693	5.444	0.001	0.48

*GNI* Gross national income per capita, PPP (current international $), 2005–2012, *GII* Gender inequality index (value: 0 = women and men equally, to 1 = women poorly), 2000–2010, *DCI* Depth of credit information index caused by financial crisis, (0 = low, to 6 = high), 2004–2012, *OLE* older age life expectancy, at age 60 years, 2000–2012

To investigate the direct relationship between OLE and GNI, GII, or DCI, a multiple regression analysis was conducted (Table 3). Regression analysis of socio-ecological indicators revealed the strongest predictors in hierarchal linear regression models (Table 4). Significant predictors of OLE in univariate analysis were used to build a model for multivariate analysis to predict OLE. Finally, higher GNI and DCI but lower GII were found to be predictors of OLE level (women and men) (R^2^ = 0.804, *p* < 0.001).

**Table 3:** Multiple regression models for predicting OLE

Variable		Coefficient	T-Value	*P*-Value	R^2^
OLE (women and men)	GNI	0.54	3.89	0.001	0.76
	GII	−0.39	−2.84	0.008	
	GII	−0.59	−5.34	0.001	0.74
	DCI	0.38	3.41	0.002	
	DCI	0.32	2.92	0.001	0.76
	GNI	0.64	5.84	0.007	
OLE (men)	GNI	0.54	3.415	0.002	0.69
	GII	−0.34	−2.197	0.036	
	GII	−0.5	−4.32	0.001	0.71
	DCI	0.45	3.84	0.001	
	DCI	0.39	4.88	0.002	0.73
	GNI	0.56	3.38	0.001	
OLE (women)	GNI	0.46	3.14	0.004	0.73
	GII	−0.45	−3.13	0.004	
	GII	−0.6	−5.41	0.001	0.73
	DCI	0.36	3.23	0.003	
	DCI	0.33	2.78	0.009	0.72
	GNI	0.6	5.05	0.001	

*GNI* Gross national income per capita, PPP (current international $), 2005–2012, *GII* Gender inequality index (value: 0 = women and men equally, to 1 = women poorly), 2000–2010, *DCI* Depth of credit information index caused by financial crisis, (0 = low, to 6 = high), 2004–2012, *OLE* older age life expectancy, at age 60 years, 2000–2012

**Table 4:** Hierarchal linear regression models for predicting OLE

Variables		Regression 1	Regression 2	Regression 3
OLE (women and men)	GNI	0.834 ***	0.535 ***	0.421**
	GII		−0.391 **	−0.327*
	DCI			0.271*
	R^2^	0.69	0.76	0.8
	Adjusted R^2^	0.69	0.74	0.78
OLE (men)	GNI	0.798***	0.535**	0.386**
	GII		−0.344*	−0.263
	DCI			0.352*
	R^2^	0.64	0.69	0.76
	Adjusted R^2^	0.63	0.67	0.74
OLE (women)	GNI	0.803***	0.456**	0.341**
	GII		−0.454**	−0.391*
	DCI			0.274**
	R^2^	0.64	0.73	0.78
	Adjusted R^2^	0.63	0.71	0.75

*p < 0.05; **p < 0.01; ***p < 0.001

*GNI* Gross national income per capita, PPP (current international $), 2005–2012, *GII* Gender inequality index (value: 0 = women and men equally, to 1=women poorly), 2000–2010, *DCI* Depth of credit information index caused by financial crisis, (0 = low, to 6=high), 2004–2012, *OLE* older age life expectancy, at age 60 years, 2000–2012

Thus, the greater the country-level OLE value, the greater its effect on increasing GNI and DCI but decreasing GII in Europe. This result indicates that OLE has significant impact on corresponding country-level GNI, GII, and DCI. The country with higher OLE is expected to have higher GNI and DCI but lower GII.

## Discussion

In this study, whether international differences in OLE level were associated with GNI per capital, GII, and DCI levels caused by financial crisis were investigated. Our results revealed that countries with high OLE level not only had higher GNI and DCI, but also had lower GII.

Lower level OLE increased socio-ecological inequalities, coinciding with unequal national income distribution between wealthy and poor countries. Country-level GNI, GII, and DCI were significantly improved over the years. However, they have not led to income, gender, or financial equity at global level. Disadvantages in gender equity, national income, and financial status are primary sources of inequality. Inequalities in national gender equity and financial level often have negative repercussions for individuals’ development [38]. Thus, associations between OEL and socio-ecological inequality prediction variables **(**GII, GNI, and DCI levels) caused by financial crisis were examined in this study to confirm if lower OEL was disproportionately susceptible.

According to extant literature, socio-ecological inequality indicators can contribute to a financial crisis [5, 10, 12, 15, 31, 32]. Results of this study revealed that increases in country-level OLE levels could lead to increases in national DCI and GNI but decreases in national GII values, suggesting that OLE can be influenced by country-level GNI, DCI, and GII.

In the current study, OLE values were the lowest in countries with low credit rating and national income. They were the highest in those with high credit rating and national income. Conversely, GII values were the highest in countries with low credit rating and national income. They were the lowest in those with high credit rating and national income. This study showed that a country’s high socio-ecological inequality indicators (e.g. GNI, GII, and DCI) could lead to low OLE levels.

Results of this study also showed that influence of GNI, GII, and DCI on OLE varied by countries. In other words, if factors associated with country-level DCI are improved with an economy recovering from a financial crisis such as that in Europe in a relatively short period of time, OLE is also increased. Thus, a financial crisis is also a crucial determinant of national-level health inequalities. This is particularly important because of the association between country-level DCI and health levels. Furthermore, in countries with a low DCI level, a financial crisis can disproportionately affect vulnerable populations in the society. It can cause a spike in suicide and death rates owing to mental and behavioural disorders, especially among those who have lost their jobs, houses, or businesses as a result [15].

In addition, many older aged ones are living on an income which may not be enough to support a healthy life. This has important implications for demand for health, residential, and social services [45]. Furthermore, our results revealed that morbidity rates among men were increased during economic turmoil and low national income, consistent with results of previous studies [3, 10, 12, 32] (see Tables 3 and 4). This could result in an increase in healthcare needs and demand for public services, colliding with austerity and privatization policies that could expose the population to further health risks [12]. Although recessions might pose risks to health, the interaction of fiscal austerity with economic shocks and weak social protection might escalate health and social crises in Europe [5].

It is known that the trend of increased longevity can significantly disrupt psycho-social and socio-economic balance between working age population and retired elderly people [46]. When countries with low-level DCI were compared to countries with high levels of DCI, the trend of increased longevity before recession might disproportionately increase the demand for medical care and the burden of families to support their elderly family member during recession.

The high correlation of OLE with GNI, GII, and DCI found in this study has a bearing on economic policies since these variables reflect government’s investment in health infrastructure. Consequently, high OLE levels are likely to contribute to high GNI and DCI but low GII. They indirectly reflect a country’s gender equality because women can become more vulnerable to stress-related illness [47]. In addition, economic change and employment could mitigate some harmful health effects of economic downturns [48]. Therefore, economic level indicators are required for a healthy living. Thus, OLE level seems to have important and latent effect on GNI, GII, and DCI. It can improve health and social health status of the elderly in a country.

However, this study has some limitations regarding the accuracy of OLE, GNI, GII, and DCI due to insufficient data from 2000 to 2012. Nevertheless, this study used the mean rate of a change index by considering time and outcome. For example, growth rate or rate of decline was used to measure the change in indicators. In this study, considerations for socio-ecological differences were based on observation of one dependent variable. Hence reductionism cannot be excluded from study conclusions. In addition, this study seems to be a cross-sectional analysis. However, it could be considered as a longitudinal analysis because mean value of 2000 to 2012 was included.

This study hypothesized that association of OLE with GNI per capital, GII value, and DCI caused by financial crisis and socio-ecological inequality indicators could help predict the health level of a population. In the three models proposed, it was evident that if countries improved their OLE values, they would obtain higher GNI and DCI but lower GII. Therefore, policies aimed at improving OLE levels are expected to have latent effects in increasing GNI and DCI but decreasing GII in European countries. Therefore, findings of this study have implications for European countries to develop successful aging strategies considering the influence of socio-ecological inequality indicators.

## Conclusions

Socio-ecological inequality levels appear to have an important latent effect on OLE levels at age 60 years in Europe. One important finding of this study in terms of relationship with older age life expectancy is that higher GNI and DCI after financial crises but smaller GII have greater effect on OLE. In particular, OLE of women was much higher than that of men. National income, gender inequality, and sovereign credit rating seem to have crucial effect on OLE in Europe. Thus, country-level strategies of successful aging in Europe should consider targeting country-level socio-ecological inequality factors such as GNI, GII, and DCI.

## Additional file

Additional file 1: Socio-ecological approach for older age life expectancy in Europe. (XLSX 10 kb)

## Abbreviations

DCI: Depth of credit information index
GII: Gender inequality index
GNI: Gross national income per capita
OLE: Old age life expectancy

## References

[1] World Health Organization, Regional Office for Europe. Healthy ageing. http://www.euro.who.int/en/health-topics/Life-stages/healthy-ageing/healthy-ageing. Accessed 16 June 2017.

[2] Mathers CD, Sadana R, Salomon JA, Murray CJ, Lopez AD. Healthy life expectancy in 191 countries, 1999. Lancet. 2001;357(9269):1685–91. 10.1016/S0140-6736(00)04824-8 11425392

[3] Kim JI, Kim G. Factors affecting the survival probability of becoming a centenarian for those aged 70, based on the human mortality database: income, health expenditure, telephone, and sanitation. BMC Geriatr. 2014a;14:113. 10.1186/1471-2318-14-113 PMC4216852 25332111

[4] Jagger C, Gillies C, Moscone F, Cambois E, Van Oyen H, Nusselder W, Robine JM, EHLEIS team. Inequalities in healthy life years in the 25 countries of the European Union in 2005: a cross-national meta-regression analysis. Lancet. 2008;372(9656):2124–31. 10.1016/S0140-6736(08)61594-9 19010526

[5] Karanikolos M, Mladovsky P, Cylus J, Thomson S, Basu S, Stuckler D, Mackenbach JP, McKee M. Financial crisis, austerity, and health in Europe. Lancet. 2013;381(9874):1323–31. 10.1016/S0140-6736(13)60102-6 23541059

[6] Mathers CD, Stevens GA, Boerma T, White RA, Tobias MI. Causes of international increases in older age life expectancy. Lancet. 2015;385(9967):540–8. 10.1016/S0140-6736(14)60569-9 25468166

[7] Stuckler D, Basu S, McKee M, Suhrcke M. Responding to the economic crisis: a primer for public health professionals. J Public Health (Oxf). 2010;32(3):298–306. 10.1093/pubmed/fdq060 20729376

[8] World Bank. Depth of credit information index, (0 = low to 8 = high). http://data.worldbank.org/indicator/IC.CRD.INFO.XQ/countries/1W?display=default. Accessed 16 June 2015a.

[9] LaVeist TA, Gaskin D, Richard P. Estimating the economic burden of racial health inequalities in the United States. Int J Health Serv. 2011;41(2):231–8. 10.2190/HS.41.2.c 21563622

[10] Bennett JE, Li G, Foreman K, Best N, Kontis V, Pearson C, Hambly P, Ezzati M. The future of life expectancy and life expectancy inequalities in England and Wales: Bayesian spatiotemporal forecasting. Lancet. 2015;386(9989):163–70. 10.1016/S0140-6736(15)60296-3 PMC4502253 25935825

[11] Williams DR, Jackson PB. Social sources of racial disparities in health. Health Aff (Millwood). 2005;24(2):325–34. 10.1377/hlthaff.24.2.325 15757915

[12] Kondilis E, Giannakopoulos S, Gavana M, Ierodiakonou I, Waitzkin H, Benos A. Economic crisis, restrictive policies, and the population's health and health care: the Greek case. Am J Public Health. 2013;103(6):973–9. 10.2105/AJPH.2012.301126 PMC3698730 23597358

[13] Tapia Granados JA, Rodriguez JM. Health, economic crisis, and austerity: A comparison of Greece, Finland and Iceland. Health Policy. 2015;119(7):941–53. 10.1016/j.healthpol.2015.04.009 25979416

[14] Walberg P, McKee M, Shkolnikov V, Chenet L, Leon DA. Economic change, crime, and mortality crisis in Russia: regional analysis. BMJ. 1998;317(7154):312–8. 10.1136/bmj.317.7154.312 PMC28623 9685275

[15] De Vogli, R. The financial crisis, health and health inequities in Europe: the need for regulations, redistribution and social protection. Int J Equity Health. 2014;13:58. 10.1186/s12939-014-0058-6 PMC4222559 25059702

[16] Asgeirsdóttir TL, Ragnarsdóttir DO. Health-income inequality: the effects of the Icelandic economic collapse. Int J Equity Health. 2014;13:50. 10.1186/1475-9276-13-50 PMC4119249 25063235

[17] Jakovljevic M, Vukovic M, Fontanesi J. Life expectancy and health expenditure evolution in Eastern Europe—DiD and DEA analysis. Expert Rev Pharmacoecon Outcomes Res. 2016;16(4):537–46. 10.1586/14737167.2016.1125293 26606654

[18] Kim JI, Kim G. Labor force participation and secondary education of gender inequality index (GII) associated with healthy life expectancy (HLE) at birth. Int J Equity Health. 2014b;13:106. 10.1186/s12939-014-0106-2 PMC4240826 25403614

[19] Kim JI, Kim G. Social Structural Influences on Healthy Aging: Community-Level Socioeconomic Conditions and Survival Probability of Becoming a Centenarian for Those Aged 65 to 69 in South Korea. Int J Aging Hum Dev. 2015;81(4):241–59. 10.1177/0091415015623550 26769915

[20] Kim JI, Kim G. Relationship Between the Remaining Years of Healthy Life Expectancy in Older Age and National Income Level, Educational Attainment, and Improved Water Quality. Int J Aging Hum Dev. 2016;83(4):402–17. 10.1177/0091415016657560 27388888

[21] Kim JI, Kim G. Country-level socioeconomic indicators associated with survival probability of becoming a centenarian among older European adults: gender inequality, male labor force participation, and proportions of women in parliaments. J Biosoc Sci. 2017;49(2):239–50. 10.1017/S0021932016000092 27071450

[22] Rowe JW, Kahn RL. Human aging: usual and successful. Science. 1987;237(4811):143–9. 10.1126/science.3299702 3299702

[23] Candore G, Balistreri CR, Listì F, Grimaldi MP, Vasto S, Colonna-Romano G, Franceschi C, Lio D, Caselli G, Caruso C. Immunogenetics, gender, and longevity. Ann N Y Acad Sci. 2006;1089:516–37. 10.1196/annals.1386.051 17261795

[24] Baker J, Meisner BA, Logan AJ, Kungl AM, Weir P. Physical activity and successful aging in Canadian older adults. J Aging Phys Act. 2009;17(2):223–35. 10.1123/japa.17.2.223 19451670

[25] Hsu HC. Impact of morbidity and life events on successful aging. Asia Pac J Public Health. 2011;23(4):458–69. 10.1177/1010539511412575 21727083

[26] Kim JI. Social factors associated with centenarian rate (CR) in 32 OECD countries. BMC Int Health Hum Rights. 2013;13:16. 10.1186/1472-698X-13-16 PMC3599594 23497053

[27] Martín U, Esnaola S. Changes in social inequalities in disability-free life expectancy in Southern Europe: the case of the Basque Country. Int J Equity Health. 2014;13(1):74. 10.1186/s12939-014-0074-6 PMC4169635 25242012

[28] Wohland P, Rees P, Nazroo J, Jagger C. Inequalities in healthy life expectancy between ethnic groups in England and Wales in 2001. Ethn Health. 2015;20(4):341–53. 10.1080/13557858.2014.921892 PMC4648377 24897306

[29] Szwarcwald CL, da Mota JC, Damacena GN, Pereira TG.Health inequalities in Rio de Janeiro, Brazil: lower healthy life expectancy in socioeconomically disadvantaged areas. Am J Public Health. 2011;101(3):517–23. 10.2105/AJPH.2010.195453 PMC3036699 21233437

[30] White C, Edgar G. Inequalities in healthy life expectancy by social class and area type: England, 2001-03. Health Stat Q. 2010 Spring;(45):28–56. 10.1057/hsq.2010.3 20383164

[31] Lee BX, Marotta PL, Blay-Tofey M, Wang W, de Bourmont S.Economic correlates of violent death rates in forty countries, 1962-2008: A cross-typological analysis. Aggress Violent Behav. 2014;19(6):729–37. 10.1016/j.avb.2014.09.016 PMC4447485 26028985

[32] Haw C, Hawton K, Gunnell D, Platt S. Economic recession and suicidal behaviour: Possible mechanisms and ameliorating factors. Int J Soc Psychiatry. 2015;61(1):73–81. 10.1177/0020764014536545 24903684

[33] Azar C, Holmberg J, Lindgren K. Socio-ecological indicators for sustainability. Ecological economics. 1996;18(2):89–112.

[34] McLeroy KR, Bibeau D, Steckler A, Glanz K. An ecological perspective on health promotion programs. Health Educ Q. 1988 Winter;15(4):351–77. 10.1177/109019818801500401 3068205

[35] National Association of Student Personnel Administrators (NASPA). Leadership for a healthy campus: an ecological approach for student success, 2004. http://www.naspa.org/help/index.cfm. Accessed 08 June 2017.

[36] Robinson T. Applying the socio-ecological model to improving fruit and vegetable intake among low-income African Americans. J Community Health. 2008;33(6):395–406. 10.1007/s10900-008-9109-5 18594953

[37] World Health Organization. Life expectancy at birth. In: Global Health Observatory Data Repository. http://apps.who.int/gho/data/view.main.680?lang=en. Accessed 16 Feb 2015.

[38] World Bank. Getting credit methodology, Depth of credit information index. In: Doing Business. http://www.doingbusiness.org/methodology/getting-credit. Accessed 16 Jun 2015b.

[39] United Nations. Gender inequality index (value). In: Human development report. http://hdr.undp.org/en/content/gender-inequality-index. Accessed 16 Feb 2015.

[40] World Bank. World Bank, International Comparison Program database. GNI per capita, PPP (current international $), http://data.worldbank.org/indicator/NY.GNP.PCAP.PP.CD?view=chart. Accessed 16 Jan 2016.

[41] United Nations. Human Development Report, Gender Inequality Index. http://hdr.undp.org/en/statistics/gii/. Accessed 15 Aug 2016: Comments and limitations. http://mdgs.un.org/unsd/mdg/Metadata.aspx?IndicatorId=0&SeriesId=557. Accessed 28 July 2016.

[42] Rahi M. Human development report 2010: Changes in parameters and perspectives. Indian J Public Health. 2011;55(4):272–5. 10.4103/0019-557X.92404 22298136

[43] Simmons BE. Average rate of change. http://www.mathwords.com. Accessed 02 June 2015.

[44] Lenzen M. Decomposition analysis and the mean-rate-of-change index. Applied Energy. 2006;83(3):185–98.

[45] O'Sullivan J, Ashton T. A minimum income for healthy living (MIHL)–older New Zealanders. Ageing & Society. 2012;32(5):747–68.

[46] Jakovljevic M, Laaser U. Population aging from 1950 to 2010 in seventeen transitional countries in the wider region of South Eastern Europe (Original research). SEEJPH 2015, posted: 21 February 2015. doi:10.12908/SEEJPH-2014-42

[47] Baruch GK, Biener L, Barnett RC. Women and gender in research on work and family stress. Am Psychol. 1987;42(2):130–6. 10.1037//0003-066x.42.2.130 3578991

[48] Stuckler D, Basu S, Suhrcke M, Coutts A, McKee M. The public health effect of economic crises and alternative policy responses in Europe: an empirical analysis. Lancet. 2009;374(9686):315–23. 10.1016/S0140-6736(09)61124-7 19589588

